# 

**Published:** 1856-04-01

**Authors:** 


### TO CORRESPONDENTS.

**We have embodied in our Retrospect the judicial psychology of the quarter. In consequence of the length of the article on "Moral and Criminal Epidemics," we have been obliged to postpone until July all our reviews, and the communication on Idiocy by the Editor.**

